# Laparoscopic surgery in a patient with foramen of Winslow hernia due to large uterine fibroids: a case report and literature review

**DOI:** 10.1186/s40792-021-01162-2

**Published:** 2021-03-25

**Authors:** Shusaku Honma, Takenori Itohara, Seigo Sha, Hirohiko Onoyama

**Affiliations:** 1 Department of Surgery, Kobe City Medical Center West Hospital, 2-4, Ichibancho, Nagataku, Kobe, Hyogo 653-0013 Japan; 2 Department of General Medicine, Nozaki Tokushukai Hospital, 2-10-5, Tanigawa, Daito, Osaka, 574-0074 Japan

**Keywords:** Foramen of Winslow hernia, Internal hernia, Laparoscopy, Uterine fibroid

## Abstract

**Background:**

Foramen of Winslow hernia (FWH) is a rare but emergent condition caused by an increase in the foramen diameter, visceral mobility, and intra-abdominal pressure. To the best of our knowledge, this is the first study to report a case of FWH secondary to large uterine fibroids that was successfully treated with laparoscopic surgery.

**Case presentation:**

A 52-year-old woman with large uterine fibroids was diagnosed with FWH. Because of the absence of signs of bowel ischemia and peritonitis, we performed an elective laparoscopic surgery through a 5-port system after bowel decompression using a long intestinal tube. Although foramen of Winslow closure was not performed, her postoperative course was uneventful.

**Conclusions:**

Laparoscopic surgery for FWH is considered an extremely effective surgical treatment option because of its safety and efficacy in performing delicate procedures (such as adhesiolysis) with a good magnified field of view.

## Background

The foramen of Winslow is the entryway to the lesser sac located anterior to the inferior vena cava and posterior to the hepatoduodenal ligament [1]. Due to various factors, abdominal contents may protrude through this opening, which results in a foramen of Winslow hernia (FWH). The incidence of FWH is rare, constituting approximately 8% of internal hernia cases [2]. Although the exact mechanism underlying the pathogenesis of FWH remains unclear, recent reports have shown that increase in the foramen of Winslow diameter (> 3 cm), visceral mobility, and intra-abdominal pressure predisposes patients to FWH [1–5]. Excessive viscera mobility may be due to long bowel mesentery, persistence of the ascending mesocolon, bowel malrotations, and large right hepatic lobe [3]. The build-up of intra-abdominal pressure may occur during pregnancy or immediate postprandial states [3].

The most common herniated organ is the small intestine (60–70%), followed by the terminal ileum, cecum, and ascending colon (25–30%) [1]. Although herniation of the gallbladder and transverse colon is extremely rare, a few studies have reported these cases [4, 6–8].

Since FWH often develops rapidly with signs of bowel strangulation and ischemia, urgent surgical treatment is required. Through increasing technological innovation, laparoscopic surgery has been widely used for FWHs [3, 9, 10].

To the best of our knowledge, no other study has reported a case of FWH caused by uterine fibroids. Here we report a case of FWH secondary to large uterine fibroids that was successfully treated with elective laparoscopic surgery under preoperative bowel decompression using a long intestinal tube.

## Case presentation

**Fig. 1:**
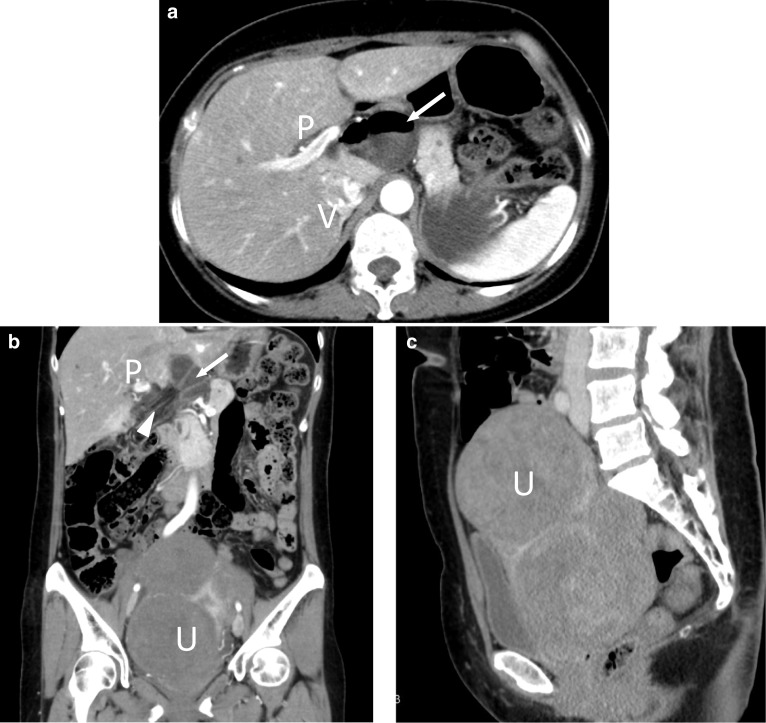
Abdominal contrast-enhanced computed tomography. **a** Axial image shows the dilated small bowel loop (arrow) between the portal vein (P) and the inferior vena cava (V). **b** Coronal image shows stretched and converging mesenteric vessels toward the foramen of Winslow (arrowheads). There are large uterine fibroids in the pelvis (U). Arrow and P indicate small bowel loops and portal vein, respectively. **c** Sagittal image shows the large uterine fibroids that are huge enough to occupy the pelvis (U)

A 52-year-old woman with unremarkable medical and surgical history presented to the emergency department with intermittent abdominal pain. Physical examinations revealed a soft abdomen with epigastric tenderness without signs of peritonitis. A gently uplifting mass from above the pubis to below the umbilicus was palpated. The patient’s white blood cell count was slightly increased to 12,220 cells/μL (normal value: 4000–8000 cells/μL), and other serum tests (e.g., C-reactive protein and liver enzymes) were within respective reference ranges. Abdominal contrast-enhanced CT showed the presence of large uterine fibroids in the pelvis and herniation of dilated small bowel loops between the portal vein and the inferior vena cava (Fig. 1).

No signs of bowel ischemia or peritonitis were observed. Because abdominal pain was refractory to previously administered medications, we performed elective laparoscopic surgery after bowel decompression using a long intestinal tube on the 19th day of hospitalization.

**Fig. 2:**
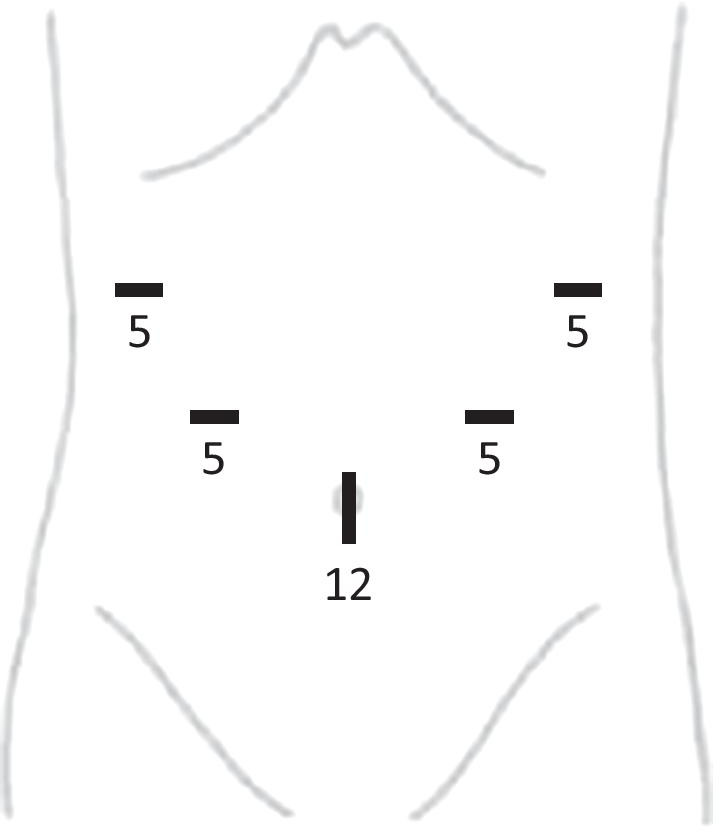
A schema of laparoscopic ports placement. A 12-mm umbilical port and four 5-mm working ports were placed in the bilateral epigastric and hypogastric regions

Laparoscopic surgery was performed using a 12-mm umbilical port and four 5-mm working ports in the bilateral epigastric and hypogastric regions (Figs. 2.and 3). We observed that the small bowel herniated through the foramen of Winslow. Hernia reduction could not be performed simply by pulling out the herniated bowel because of the adhesion between the herniated bowel and intra-omental tissue. Hence, we incised the lesser omentum to open the lesser sac and facilitate adhesiolysis.

**Fig. 3:**
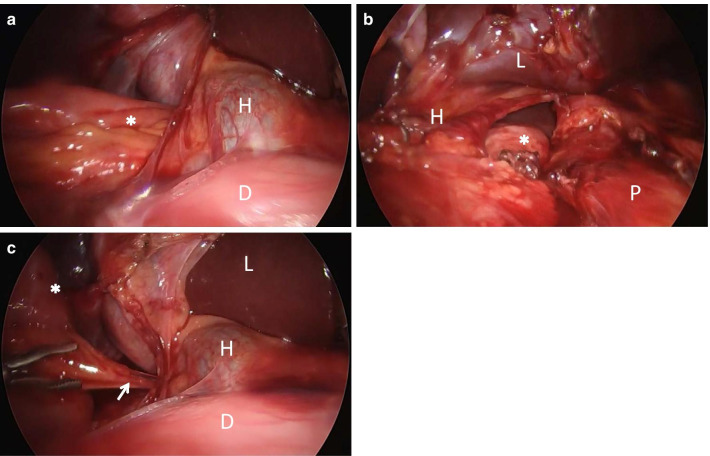
Intraoperative laparoscopic views. **a** Small bowel (*) herniated through the foramen of Winslow. The bowel dilation reduced with the effect of the nasal tube before surgery. Due to adhesion between intra-omental tissue and herniated bowel, the herniated bowel was difficult to pull out from the foramen of Winslow. **b** The view shows the lesser sac opened by incising the lesser omentum. **c** The arrow shows one of the adhesions between the intra-omental tissue and herniated bowel. Duodenum, hepatoduodenal ligament, liver, and pancreas are labeled D, H, L, and P, respectively

Because the herniated bowel was observed to be viable, bowel resection was not performed. No intestinal malrotation or long bowel mesentery was observed, and closure of the foramen of Winslow was not performed. Nevertheless, there were no postoperative complications and the patient was discharged on postoperative day 6. No recurrences have occurred after 12 months of follow-up.

## Discussion

The incidence of FWH is rare, constituting approximately 8% of internal hernia cases [2]. Although the exact mechanism underlying FWH is largely unknown, recent reports have implicated dilation of the foramen of Winslow, excessive viscera mobility (e.g., long bowel mesentery, persistence of the ascending mesocolon, and bowel malrotations), and increase in the intra-abdominal pressure (e.g., pregnancy and immediate postprandial periods) as predisposing factors for the FWH [1–5]. In this case, large uterine fibroids may have caused an increase in the intra-abdominal pressure, as in pregnancy. Currently, based on the available literature, only two FWH cases have been reported in pregnant women [5, 11]. To the best of our knowledge, this is the first case report of FWH caused by uterine fibroids. The causal correlation between large uterine fibroids and FWH remains unclear. In addition to the increase in the intra-abdominal pressure, large uterine fibroids may have pushed the small bowel out towards the upper abdomen, leading to FWH. The fact that there were some adhesions between the intra-omental tissue and the herniated bowel suggests that the small bowel, which was pushed out by large uterine fibroids, protruded through the foramen of Winslow for a long time.

Uterine fibroid is the most common type of tumor that grows in the uterus and presents with various clinical symptoms, such as heavy menstrual bleeding, iron deficiency anemia, infertility, pelvic pain, and pelvic masses [12]. For asymptomatic uterine fibroids, no intervention or further investigation is required. In contrast, for symptomatic patients, interventions (i.e., myomectomy, hysterectomy, uterine-artery embolization, or hormone therapy) are selected according to their inclinations (e.g., preserving fertility and their uterus) [13]. Because large uterine fibroids can cause internal hernias, treatment is necessary even in the absence of gynecologic symptoms.

Recent reports have revealed that laparoscopic surgery is feasible for FWH. To determine the characteristics of laparoscopic surgery for FWH, we performed a comprehensive literature search and identified 18 case reports in the field [4, 6–10, 14–25].

Table 1 outlines the characteristics of 19 cases including this case report. Although there were more female cases (*n* = 17) than male cases (*n* = 2), the incidence of FWH was higher in males than in females [2]. There were no cases of postoperative complications, including four cases wherein bowel resection was performed. In laparoscopic surgery, careful maneuver (i.e., gentle traction using atraumatic bowel graspers) is required when pulling the dilated bowel out of the foramen of Winslow [18]. Despite this, spontaneous hernia reduction was achieved at the time of surgery in some cases [22, 26]. In this case, the successful bowel decompression supposedly contributed to decreasing the risk of bowel injury. Elective laparoscopic surgery after bowel decompression using a long intestinal tube may be suitable when there are no signs of bowel ischemia or peritonitis at the time of diagnosis.

**Table 1:** Laparoscopic management of foramen of Winslow hernia

References	Year	Age/sex	Herniated organ	Bowel resection	Foramen closure	Preventive measures other than foramen closure	Postoperative complications	Postoperative hospitalization (day)	Recurrence	Follow-up duration
Izumi et al. [4]	2009	70/F	Gallbladder	No	No	Cholecystectomy	NM	NM	NM	NM
Webb et al. [9]	2009	60/F	Cecum	No	NM	NM	None	1	NM	NM
Van Daele et al. [10]	2011	40/F	Right colon	No	No	None	None	6	No	6 days
Clough et al. [6]	2011	28/F	Transverse colon	No	No	None	None	4	No	4 months
Yamashiro et al. [14]	2013	65/F	Ileum	Ileum resection	No	None	NM	NM	No	14 days
Lin et al. [15]	2013	48/F	Ileum	No	No	None	None	NM	NM	NM
May et al. [16]	2013	64/F	Small bowel	No	No	The greater omentum was used to obturate the foramen of Winslow	None	6	NM	NM
Numata et al. (7)	2013	90/F	Gallbladder	No	No	Cholecystectomy	None	17	No	17 days
Ryan et al. [17]	2014	45/F	Right colon, terminal ileum	No	No	Appendectomy	None	NM	NM	NM
Harnsberger et al. [18]	2015	57/F	Cecum, terminal ileum	Right hemicolectomy	No	The foramen of Winslow was obliterated with mobilized omentum	None	4	No	21 months
Garg et al. [19]	2016	77/M	Cecum, terminal ileum	No	Yes (silk sutures)	Cecopexy, appendectomy	None	NM	NM	NM
Daher et al. [20]	2016	38/F	Cecum, terminal ileum	No	No	Cecopexy, appendectomy	None	2	No	6 months
Duinhouwer et al. [21]	2016	32/F	Ascending colon	No	No	None	None	NM	No	6 months
Brandao et al. [8]	2016	42/F	Transverse colon	No	Yes (metal clips)	None	None	3	No	15 months
Cho et al. [22]	2017	32/F	Small bowel	Small bowel resection	No	None	None	6	No	NM
Lyons et al. [23]	2017	58/F	Cecum, terminal ileum	No	NM	NM	NM	NM	NM	NM
Ichikawa et al. [24]	2017	35/M	Small bowel	No	No	None	None	6	NM	NM
Yasir Abdu et al. [25]	2019	47/F	Cecum, terminal ileum	Ileocecal resection	No	None	None	NM	No	NM
Our case		52/F	small bowel	No	No	None	None	6	No	12 months

*F* female, *M* male, *NM* not mentioned

Foramen closure was performed in only two cases (Table 1). The opening may be closed using a mesh for inguinal hernia or abdominal wall hernia. The efficacy of foramen closure in preventing the recurrence of FWH remains debatable. Because of the risk of injury to the bile duct, portal vein, and hepatic artery [21], some surgeons have obliterated the lesser omentum rather than closing the foramen [16, 18]. Nevertheless, no recurrence was observed regardless of the performance of preventive measures. Furthermore, each predisposing factor has to be addressed to prevent a recurrence. In this case, treatment of the large uterine fibroids is the most important measure to prevent FWH recurrence.

## Conclusion

We experienced a rare case of FWH due to large uterine fibroids. Laparoscopic surgery for FWH is considered to be a very effective treatment option not only because of its minimally invasive nature, but also its utility to perform delicate procedures (such as adhesiolysis) with a good magnified field of view. Treatment of the large uterine fibroids should be considered to prevent the recurrence of FWH rather than foramen of Winslow closure.

## Data Availability

The datasets supporting the conclusions of this article are included in this paper.

## Abbreviation

FWH: Foramen of Winslow hernia
